# Weight Perceptions and Perceived Risk for Diabetes and Heart Disease Among Overweight and Obese Women, Suffolk County, New York, 2008

**DOI:** 10.5888/pcd9.110185

**Published:** 2012-04-05

**Authors:** Susan Darlow, Melody S. Goodman, Jewel D. Stafford, Christina R. Lachance, Kimberly A. Kaphingst

**Affiliations:** Cancer Prevention and Control Program, Fox Chase Cancer Center; Washington University School of Medicine, St. Louis, Missouri; Washington University School of Medicine, St. Louis, Missouri; National Human Genome Research Institute, National Institutes of Health, Bethesda, Maryland; Washington University School of Medicine, St. Louis, Missouri

## Abstract

**Introduction:**

Many Americans fail to accurately identify themselves as overweight and underestimate their risk for obesity-related diseases. The purpose of this study was to investigate associations between weight perceptions and perceived risk for diabetes and heart disease among overweight or obese women.

**Methods:**

We examined survey responses from 397 overweight or obese female health center patients on disease risk perceptions and weight perceptions. We derived odds ratios (ORs) and 95% confidence intervals (CIs) from multivariable logistic regression analyses to examine predictors of perceived risk for diabetes and heart disease. We further stratified results by health literacy.

**Results:**

Perceiving oneself as overweight (OR, 2.78; 95% CI, 1.16-6.66), believing that being overweight is a personal health problem (OR, 2.46; 95% CI, 1.26-4.80), and family history of diabetes (OR, 3.22; 95% CI, 1.53-6.78) were associated with greater perceived risk for diabetes. Perceiving oneself as overweight (OR, 4.33; 95% CI, 1.26-14.86) and family history of heart disease (OR, 2.25; 95% CI, 1.08-4.69) were associated with greater perceived risk for heart disease. Among respondents with higher health literacy, believing that being overweight was a personal health problem was associated with greater perceived risk for diabetes (OR, 4.91; 95% CI, 1.68-14.35). Among respondents with lower health literacy, perceiving oneself as overweight was associated with greater perceived risk for heart disease (OR, 4.69; 95% CI, 1.02-21.62).

**Conclusion:**

Our findings indicate an association between accurate weight perceptions and perceived risk for diabetes and heart disease in overweight or obese women. This study adds to research on disease risk perceptions in at-risk populations.

## Introduction

Diabetes and heart disease are prevalent chronic diseases in the United States. Diabetes affects 8.3% of US adults (1), heart disease, 6.5% (2). Approximately half of those diagnosed with diabetes are women (1), and more women than men die from heart disease (3).

Obesity is a contributing risk factor for both diabetes and heart disease (4). However, many US adults are not aware of this association (5) and do not consider obesity to be a serious health concern (6). Accurate weight perception is a key tool in identifying risk for obesity-related chronic disease and may encourage people to lose weight (4). Despite efforts and encouragement by physicians and other health professionals, a strong societal focus on weight loss and management, and marketing efforts to accurately assess weight and encourage weight loss, more than 75% of overweight Americans fail to accurately identify themselves as overweight or obese (7).

As with weight perceptions, many Americans tend to underestimate their risk for obesity-related chronic diseases, including diabetes and heart disease (8). Women, particularly black and Hispanic women, frequently underestimate their health risks associated with obesity (9). Higher health literacy is associated with more knowledge of chronic disease (10) and may play a role in perceived risk for chronic diseases such as diabetes and heart disease. Limited health literacy is associated with low medication adherence for chronic disease (11), heart disease deaths (12), poor glycemic control, and more diabetes-related complications (13).

Our objective was to examine predictors of perceived risk for diabetes and heart disease among overweight and obese women, specifically, perceptions about being overweight. Given the high rates of obesity among black and Hispanic women (14), study findings can contribute to targeted disease risk interventions for high-risk populations. We excluded men from our study because of sex differences in overweight and obesity; being overweight is associated with better psychosocial functioning in men but not women (15). Because people with poor health literacy might not be aware of the link between being overweight and chronic disease, we stratified analyses by health literacy. We hypothesized that perceiving oneself as overweight and believing that being overweight is a personal health problem would be associated with perceived risk for disease in women with higher but not lower health literacy.

## Methods

### Overview

We used multivariable logistic regression to assess associations between weight perceptions and perceived risk for diabetes and heart disease. The moderating role of health literacy was examined by stratifying by level of health literacy (high vs low levels). Female patients at community health centers (N = 397) completed questionnaires that assessed study variables. Data were collected between August and November 2008. This study was approved by the Stony Brook University Committee on Research Involving Human Subjects, the Suffolk County Department of Health Services institutional review board, and the National Institutes of Health Office of Human Subjects Research.

### Procedure

Suffolk County is a diverse region that encompasses the eastern two-thirds of Long Island, New York. The Suffolk County Department of Health Services is the safety-net provider of health care for county residents with Medicaid and provides low-cost or free care on a sliding scale for uninsured or underinsured patients. It has a network of 8 community health centers, typically in medically underserved areas. Trained data collectors recruited study participants in waiting rooms at all 8 health centers.

We administered paper surveys at each of the health centers on different days of the week and at different times of the day. Questionnaires were completed as part of a parent study designed to assess beliefs about heritability of disease and health behaviors among community health center patients; as part of the survey, participants were asked questions about their weight and risk perceptions. Upon completion of the survey, participants could choose from various small incentives, such as travel coffee mugs, notepads, and change purses.

### Participants

Eligibility criteria for the parent study required that participants be at least 18 years old and able to complete the survey in English or Spanish. Sixty-five percent of people approached agreed to complete the survey. Of the 1,318 who agreed to participate, 1,061 (81%) completed all components of the survey. We found no significant differences in demographic characteristics between people with complete surveys and those with incomplete surveys. The survey respondents were generally similar to the underlying patient population of the Suffolk County Department of Health Services in sex and age but had a smaller proportion of Hispanics. The sample for this study was 397 women who were classified as overweight or obese (body mass index [BMI] ≥25 kg/m^2^), based on self-reported height and weight. Participants' ages ranged from 18 to 80 (mean, 38.0; standard deviation, 13.4). We did not obtain objectively measured data on height, weight, or age.

### Measures

**Predictor variables**

Survey respondents indicated their self-perceived weight status using an item that assessed their perceptions of their weight category (underweight, normal weight, or overweight, dichotomized to overweight or not). Using similar questions to those from the National Health and Nutrition Examination Survey (16), we asked participants to indicate on a 4-point scale the degree to which they believed that being overweight was a health problem for them personally (from 1 = "not a problem" to 4 = a "big" health problem). For analysis, we dichotomized this variable to respondents who believed being overweight is a "big" health problem for them personally and those who did not. These 2 items (perceiving oneself as overweight and perceiving that being overweight is a personal health problem) assessed weight perceptions.

**Outcome variables**

We assessed perceived risk for diabetes and heart disease using 2 items ("Compared to the average person your age, how likely are you to get diabetes/heart disease?") adapted from the Health Information National Trends Survey (17). Participants responded using a 5-point Likert-type scale (from 1 = "a lot less likely" to 5 = "a lot more likely"). These 2 items were dichotomized to "low perceived risk" (score of 1, 2, or 3) and "high perceived risk" (score of 4 or 5).

**Moderating variable**

We assessed Participants' level of health literacy using the Newest Vital Sign (NVS), a 6-item instrument based on a standard food nutrition label that requires reading comprehension and numeracy skills. The NVS is available in both English and Spanish and is valid for detecting limited health literacy (18,19); it is easy to administer and takes less time to complete than other measures of health literacy (20). Participants received an NVS score ranging from 0 to 6 based on the number of correct answers. A score of 0 or 1 reflects a high likelihood of limited health literacy; 2 or 3, a possibility of limited health literacy; and 4 to 6, adequate health literacy. For this study, we defined "higher health literacy" as adequate literacy and "lower health literacy" as a high or possible likelihood of limited health literacy.

**Demographic variables**

Participants reported their race/ethnicity, sex, age, height, weight, family history of disease, and whether they had ever been diagnosed with heart disease or diabetes. Given the low numbers of Asian/Pacific Islander, Native American, and other race respondents, we limited analysis to women who identified as non-Hispanic white, non-Hispanic black, or Hispanic.

### Statistical analyses

We first conducted a series of univariate analyses to determine the distributions of the variables of interest. We conducted bivariate analyses to examine the effects of the predictors and demographic variables on both outcome variables. We then conducted multivariable logistic regression analyses to examine whether weight perception predicted perceived relative risk for diabetes and heart disease. We controlled for race/ethnicity, family history of diabetes or heart disease, and age (dichotomized at <40 or ≥40). We then examined separate multivariable logistic regression models for higher versus lower health literacy, including the same predictors and demographic variables. Participants diagnosed with diabetes or heart disease were excluded from the analyses of perceived relative risk for diabetes and heart disease, respectively. Data were analyzed using SAS version 9.2 (SAS Institute, Inc, Cary, North Carolina). Significance was set at *P* < .05.

## Results

Forty-three percent of our sample was non-Hispanic black, 30% was Hispanic, and 27% was non-Hispanic white (Table 1). Thirty-nine percent had higher health literacy. Black (χ^2^ = 4.2, *P* = .04) and Hispanic (χ^2^ = 21.6, *P* < .001) participants were more likely to have lower health literacy than were white participants.

### Perceived risk for diabetes

Perceiving oneself as overweight, believing that being overweight is a personal health problem, and family history of diabetes were associated with greater perceived risk for diabetes  (Table 2). Among participants with higher health literacy, believing that being overweight is a personal health problem and having a family history of diabetes were associated with greater perceived risk for diabetes.

### Perceived risk for heart disease

Perceiving oneself as overweight was associated with greater perceived risk for heart disease, as was family history of heart disease  (Table 3). Among women with lower health literacy, perceiving oneself as overweight and family history of heart disease were associated with greater perceived risk for heart disease.

## Discussion

Overall, beliefs about one's weight were associated with greater perceived risk for diabetes and heart disease among overweight and obese women. Although some studies have examined awareness of the link between obesity and chronic illness (5,21), to our knowledge, no other studies have examined personal weight perceptions and the association with perceived risk for diabetes and heart disease.

On the basis of previous research on health literacy, we reasoned that health literacy would be associated with greater perceived risk for diabetes and heart disease among women who are overweight or obese, which would indicate that these women accurately identify themselves as overweight and are aware that being overweight increases their risk for diabetes and heart disease. When we stratified the analyses by level of health literacy, weight perceptions were associated with perceived risk for diabetes but not heart disease among women with higher health literacy. However, participants with lower health literacy accurately identified themselves as at greater risk for heart disease based on their weight and family history. These results may indicate that people perceive diabetes and heart disease differently. The risk factors for heart disease may be better known than for diabetes because heart disease is the leading cause of death among women. Therefore, perhaps less attention is paid to diabetes, and the risk factors for this disease are less well known, which has been shown in research on perceived risk for diabetes (22,23).

It is unclear why weight perceptions were associated with perceived risk for diabetes but not heart disease among participants with higher health literacy. Given the prevalence of heart disease, perhaps people with higher health literacy educate themselves more on other risk factors for heart disease, such as high triglyceride levels and smoking (24,25). If so, people with higher health literacy may view these risk factors as having a greater effect than being overweight. People with higher health literacy also may be engaging in weight-loss behaviors to a greater extent than those with lower health literacy. A study of older adults did not find an association between health literacy and unhealthy behaviors (26), but future research should examine this possible association in a more generalizable sample. People engaging in healthy behaviors may perceive their risk for chronic disease as reduced, but additional research with a larger sample is needed.

We did not find, as another study did, that black and Hispanic women have poorer perceived risk for chronic disease than do white women (21), perhaps be because our sample came from a socioeconomically homogenous group of county health center patients. Socioeconomic status may be a greater predictor of perceived risk for chronic disease than race/ethnicity. Sociocultural differences associated with race/ethnicity should also be factored into risk and weight perceptions.

This study had several limitations. Questionnaire responses were collected from a convenience sample of low-income women from Long Island, New York. Our findings may not generalize to women from other parts of the country. We did not assess Participants' reasons for visiting the health centers, and data are based on self-reports. We attempted to address the latter issue by conducting an anonymous survey. Although people sometimes underestimate their weight, their estimates are generally accurate (27,28); however, the lack of an objective measure of BMI is a major study limitation. BMI does not assess adiposity, and being overweight but not obese, based on BMI standards, does not always result in adverse health consequences (29,30). The results from the analyses stratified by health literacy should be interpreted cautiously, given the small cell sizes. Nevertheless, stratifying the analyses by level of health literacy was preferable to treating health literacy as a covariate, given our interest in examining health literacy as a moderator.

A study strength is that we used a comprehensive definition of weight perception by including both an item about perceived weight status and an item about perception of one's weight status being a health problem. Future research should continue to examine accuracy of perceived weight status and associations with perceived risk for other conditions associated with obesity, such as hypertension and some types of cancer. Future studies should also use larger, representative samples because it is possible that some effects were undetected, given our small cell sizes. Appropriately identifying pathways that are associated with disease risk perceptions may lead to a greater understanding of other obesity-related risk factors. Health professionals should try to ensure that patients who are overweight are aware of their own weight status and of the associated health risks. Health professionals should also be aware of the role of health literacy when offering health recommendations to patients.

## Figures and Tables

**Table 1.** Self-Reported Characteristics of Overweight and Obese Female Patients at Community Health Centers, by Race/Ethnicity and Health Literacy^a^, Suffolk County, New York, 2008

**Characteristic**	Non-Hispanic Black, n (%) (n = 170)	Hispanic, n (%) (n = 118)	Non-Hispanic White, n (%) (n = 109)	Higher Literacy, n (%) (n = 140)	Lower Literacy, n (%) (n = 222)	Overall, n (%) (N = 397)
**Diabetes^b^**						
High perceived risk	29 (18.6)	27 (24.1)	32 (30.2)	28 (23.7)	27 (15.3)	88 (23.5)
Low perceived risk	127 (81.4)	85 (75.9)	74 (69.8)	90 (76.3)	149 (84.7)	286 (76.5)
Family history	111 (67.7)	77 (67.5)	63 (60.0)	86 (63.2)	141 (66.2)	251 (65.5)
No family history	53 (32.3)	37 (32.5)	42 (40.0)	50 (36.8)	72 (33.8)	132 (34.5)
**Heart disease^c^**						
High perceived risk	23 (14.2)	16 (14.2)	26 (24.5)	22 (17.5)	23 (12.5)	65 (17.1)
Low perceived risk	139 (85.8)	97 (85.8)	80 (75.5)	104 (82.5)	161 (87.5)	316 (82.9)
Family history	57 (37.0)	48 (47.5)	57 (57.6)	65 (49.6)	83 (42.6)	162 (45.8)
No family history	97 (63.0)^d^	53 (52.5)	42 (42.4)	66 (50.4)	112 (57.4)	192 (54.2)
**Age, y**						
<40	93 (56.0)	69 (61.6)^e^	51 (48.1)	80 (58.0)	112 (52.1)	213 (55.5)
≥40	73 (44.0)	43 (38.4)	55 (51.9)	58 (42.0)	103 (47.9)	171 (44.5)
**Weight perception**						
Perceives self as overweight	109 (64.5)	76 (65.0)	87 (79.8)^e^	111 (79.9)^f^	136 (61.5)	272 (68.9)
Does not perceive self as overweight	60 (35.5)	41 (35.0)	22 (20.2)	28 (20.1)	85 (38.5)	123 (31.1)
Overweight is personal health problem	89 (52.7)	64 (56.1)	52 (47.7)	68 (49.3)	119 (54.1)	205 (52.3)
Overweight is not personal health problem	80 (47.3)	50 (43.9)	57 (52.3)	70 (50.7)	101 (45.9)	187 (47.7)

a Assessed using the Newest Vital Sign instrument (18) and defined as adequate health literacy ("higher") vs any likelihood of limited health literacy ("lower"). The 2 categories do not add to the total of 397 because not all respondents took the literacy test.

b Comparison group was low perceived risk. Participants diagnosed with diabetes (n = 92) were excluded from this analysis.

c Participants diagnosed with heart disease (n = 57) were excluded from this analysis.

d *P* < .01.

e *P* < .05.

f *P* < .001.

**Table 2.** Associations Between Weight Perceptions and High Perceived Risk of Diabetes, by Health Literacy,^a^ Among Female Patients at Community Health Centers, Suffolk County, New York, 2008^b^

Characteristic	Higher Health Literacy			Lower Health Literacy			Overall		

	n	OR (95% CI)	*P* Value^c^	n	OR (95% CI)	*P* Value^c^	n	OR (95% CI)	*P* Value^c^
Non-Hispanic black	184	0.69 (0.23-2.09)	.52	122	0.37 (0.12-1.10)	.07	306	0.52 (0.25-1.09)	.08
Hispanic	184	2.93 (0.70-12.24)	.14	122	0.33 (0.10-1.11)	.07	306	0.62 (0.27-1.42)	.26
Family history of diabetes	176	4.01 (1.27-12.62)	.02	118	2.91 (1.00-8.43)	.05	294	3.22 (1.53-6.78)	.002
Age ≥40^d^	178	1.90 (0.63-5.72)	.26	121	1.20 (0.49-2.93)	.68	299	1.18 (0.62-2.26)	.61
Perceives self as overweight	183	3.03 (0.57-16.21)	.19	121	2.29 (0.78-6.69)	.13	304	2.78 (1.16-6.66)	.02
Overweight is personal health problem	182	4.91 (1.68-14.35)	.004	120	1.71 (0.68-4.32)	.25	302	2.46 (1.26-4.80)	.01

Abbreviations: OR, odds ratio; CI, confidence interval.

a Assessed using the Newest Vital Sign instrument (18) and defined as adequate health literacy ("higher") vs any likelihood of limited health literacy ("lower").

b Comparison group was low perceived risk. Participants diagnosed with diabetes (n = 52) were excluded from this analysis.

c Calculated by using χ^2^ test.

d Dichotomized to <40 y and ≥40 y.

**Table 3.** Associations Between Weight Perceptions and High Perceived Risk of Heart Disease, by Health Literacy,^a^ Among Female Patients at Community Health Centers, Suffolk County, New York, 2008^b^

**Characteristic**	Higher Health Literacy			Lower Health Literacy			Overall		

	n	OR (95% CI)	*P* Value^c^	n	OR (95% CI)	*P* Value^c^	n	OR (95% CI)	*P* Value^c^
NonHispanic black	201	0.68 (0.22-2.09)	.50	129	0.86 (0.21-3.47)	.83	330	0.69 (0.30-1.57)	.37
Hispanic	201	0.30 (0.03-2.66)	.28	129	0.95 (0.23-3.89)	.95	330	0.60 (0.24-1.53)	.28
Family history of heart disease	175	1.50 (0.52-4.33)	.46	121	3.31 (1.17-9.35)	.02	296	2.25 (1.08-4.69)	.03
Age ≥40^d^	195	2.08 (0.73-5.97)	.17	127	1.47 (0.53-4.04)	.46	322	1.71 (0.83-3.51)	.14
Perceives self as overweight	200	3.53 (0.41-30.55)	.25	129	4.69 (1.02-21.62)	.047	329	4.33 (1.26-14.86)	.02
Overweight is personal health problem	199	2.25 (0.75-6.74)	.15	127	1.37 (0.48-3.92)	.56	326	1.87 (0.90-3.91)	.10

Abbreviations: OR, odds ratio; CI, confidence interval.

a Assessed using the Newest Vital Sign instrument (18) and defined as adequate health literacy ("higher") vs any likelihood of limited health literacy ("lower").

b Participants diagnosed with heart disease (n = 29) were excluded from this analysis.

c Calculated by using χ^2^ test.

d Dichotomized at <40 y and ≥40 y.
